# Parental perceptions of body weight and appetite in infants and toddlers with cystic fibrosis

**DOI:** 10.1016/j.appet.2024.107357

**Published:** 2024-07-01

**Authors:** Sarah Ann Duck, Elena Jansen, Afroditi Papantoni, Aerial Sheltry, Daphne Koinis-Mitchell, Viren D’Sa, Sean Deoni, Timothy H. Moran, Robert L. Findling, Peter J. Mogayzel, Susan Carnell

**Affiliations:** a Division of Child and Adolescent Psychiatry, Department of Psychiatry and Behavioral Sciences, Johns Hopkins University School of Medicine, Baltimore, MD, USA; b Eudowood Division of Pediatric Respiratory Sciences, Department of Pediatrics, Johns Hopkins Medicine, USA; c Department of Behavioral Psychology, Kennedy Krieger Institute, Baltimore, MD, USA; d Department of Psychiatry and Behavioral Sciences, Johns Hopkins University School of Medicine, USA; e Department of Psychiatry, Virginia Commonwealth University School of Medicine, Richmond, VA, USA; f Department of Pediatrics, Warren Alpert Medical School at Brown University, Providence, RI, USA; g Department of Psychology, Social Sciences, University of Stavanger, Stavanger, Norway; h Bradley-Hasbro Children's Research Center, Rhode Island Hospital, Providence, RI, USA

**Keywords:** Infants, Cystic fibrosis, Appetite, Appetitive traits, perceived weight, parent feeding

## Abstract

Nutritional status has clinical relevance and is a target of guidance to parents of children with cystic fibrosis (CF). Growth is routinely monitored in CF clinics but there is no standardized way of assessing appetitive behaviors or parents' perceptions of their children's appetite. Greater understanding of these factors could improve clinical guidance regarding parent feeding behaviors. We therefore aimed to assess parent perceptions of child weight, and parent reports of child appetite using the Baby Eating Behavior Questionnaire (BEBQ), in a sample of infants and toddlers with CF, compared with a community sample. We additionally assessed relationships of parent perceptions of child weight with parent feeding behaviors in the sample with CF. Anthropometric and questionnaire data were collected for 32 infants and toddlers with CF, as well as 193 infants and toddlers drawn from RESONANCE, a community cohort study. Parents perceived children with CF to be lower in weight than their actual weight, to a greater extent than was evident in the community sample. Parents who perceived their children with CF to be underweight vs. right weight reported greater slowness in eating on the BEBQ. Parents perceived children with CF to have greater slowness in eating and lower enjoyment of food, compared to parents of children in the community sample, independent of sample differences in child weight, age, and sex. Our results demonstrate the potential utility of the BEBQ in a clinical sample and suggest it may be helpful for clinicians to assess parents' perceptions of their child's weight and appetite to promote a fuller understanding of the child's nutritional status, facilitate appropriate feeding behaviors and alleviate unnecessary concerns.

## 1 Introduction

Malnutrition is a prevalent issue in children and adults with cystic fibrosis (CF) (Cystic Fibrosis Foundation, 2011). Slow growth velocity and reduced weight is common (Stallings, Stark, Robinson, Feranchak, & Quinton, 2008) due to malabsorption and elevated metabolism caused by increased respiratory workload and inflammation. Higher body mass index (BMI) values are associated with improved lung health in those with CF (Konstan et al., 2003; Regelmann et al., 2013; Steinkamp & Wiedemann, 2002), making maintenance of a healthy rather than a low weight an essential health goal in the CF population. Highly effective CFTR modulator therapies have been transformative in improving health for patients with CF (Jia & Taylor-Cousar, 2023), leading to a reduction in malnutrition and underweight in both children and adults with CF and raising potential concerns about the health implications of overweight, a previously uncommon occurrence (Gabel et al., 2022). However, these drugs are not currently available or approved for use in majority of children <2 years old with CF. Therefore, malnutrition remains a critical concern for these children.

Given the importance of nutritional status to health outcomes, parents of children with CF have reported greater concern regarding potential feeding problems than parents of healthy children (Sanders, Turner, Wall, Waugh, & Tully, 1997). During interviews, parents of children 6–14 years old with CF express that keeping their child's weight up to protect their health is a main priority of feeding, and that their child's cooperation with eating is a challenge during mealtimes (Savage & Callery, 2005). Parents of children with CF have also been observed to use an increased frequency of control behaviors (e.g. prompting, coaxing, commanding) in order to address eating difficulties in their children (Stark & Powers, 2005). However, while the importance of eating behaviors is established in children and adults with CF, research regarding appetitive characteristics-enduring dispositions towards food (food approach and food avoidance tendences; (Carnell & Wardle, 2008) that have been shown in healthy children to track through development (Ashcroft, Semmler, Carnell, van Jaarsveld, & Wardle, 2008; Jansen, Thapaliya, et al., 2023) and show genetic influence (Carnell, Haworth, Plomin, & Wardle, 2008; Llewellyn, Van Jaarsveld, Johnson, Carnell, & Wardle, 2010)- is relatively underdeveloped, and there is no consensus on an objective method of assessing appetite in CF (McTavish & Thornton, 2022). In a previous study we validated the Child Eating Behavior Questionnaire (CEBQ; Carnell & Wardle, 2007; Wardle, Guthrie, Sanderson, & Rapoport, 2001) in parents of children aged 2–12 years with CF and found that children with CF had lower scores on appetite in association with lower weight, and as compared to children (Papantoni et al., 2019). However, this study focused on children from 2 to 12 years old and did not assess appetite in infancy, during the milk-feeding stage. Further, there is evidence that parents' perceptions of their child's weight may not always be correct, even within non-clinical populations. For example, a study of healthy, Australian toddlers (12–16 months) found that lean, but healthy-weight toddlers, were often perceived as underweight by their mothers, while, among the children with overweight, only the heaviest were perceived as overweight (Byrne, Magarey, & Daniels, 2016). Parental perceptions of weight may therefore be particularly inaccurate for infants and toddlers with CF, among whom feeding and growth are of high relevance to health.

The main aims of the current study were: (i) to test the reliability and validity of the Baby Eating Behavior Questionnaire (BEBQ; Llewellyn, van Jaarsveld, Johnson, Carnell, & Wardle, 2011), a parent-report measure of infant appetite that shows associations with body weight in healthy children (Kininmonth et al., 2021), in a sample of young children with CF, (ii) to examine relationships of parents' perceptions of their child's weight with their actual weight in infants and toddlers with CF as well as a community sample, (iii) to evaluate relationships of parents' child weight perceptions with parent-reported appetite and parent feeding behaviors in infants and toddlers with CF, and (iv) to compare parents' perception of children's appetitive characteristics between children with CF and a community sample. We hypothesized that (i) the BEBQ would show good reliability and validity in the current study population, (ii) parents of young children with CF would misperceive their children as underweight, to a greater extent than in the community sample, (iii) infants and toddlers with CF who were perceived to be underweight vs. right weight would have higher scores on food avoidance subscales and parental pressure to eat and monitoring, and lower scores on food approach subscales and parental restrictive feeding, and (iv) infants and toddlers with CF would have higher scores on food avoidance subscales and lower scores on food approach subscales compared with children from the community sample.

## 2 Methods

Parents of infants and toddlers (<2 years old) with CF were invited to participate during a routine visit to the Johns Hopkins Pediatric CF Clinic. This study was approved by the Johns Hopkins Institutional Review Board in accordance with the Declaration of Helsinki (IRB Approval Number: IRB0055313), and parents provided written informed consent. We collected data on height and weight measured by the clinic staff. Parents completed the BEBQ, Child Feeding Questionnaire (CFQ), a medication survey, and a question on current feeding method. Additional clinical data (genotype, pancreatic function, presence of gastric tube) and demographic data (age, ethnicity, race, sex) was extracted from the electronic medical record. Pancreatic insufficiency was defined by the use of pancreatic enzyme supplementation.

### 2.1 Measures

#### 2.1.1 Baby eating questionnaire (BEBQ)

The Baby Eating Behavior Questionnaire (BEBQ; Llewellyn et al., 2011) is an 18-item parent-report questionnaire used to assess appetitive characteristics of young children. The BEBQ is derived from the validated, widely used CEBQ and has been psychometrically validated in healthy populations (Hunot-Alexander et al., 2021; Oyama et al., 2021). The BEBQ comprises a single-item construct General Appetite (e.g., My baby had a big appetite) as well as four subscales. These subscales - Enjoyment of Food (e.g., My baby enjoyed feeding time), Food Responsiveness (e.g., If given the chance, my baby would always be feeding), Slowness in Eating (e.g., My baby fed slowly), and Satiety Responsiveness (e.g., My baby found it difficult to manage a complete feed) have demonstrated good internal consistency in healthy infants (Llewellyn et al., 2011). Item responses are given on a 5-point Likert scale ranging from Never to Always. The food approach subscales- Food Responsiveness and Enjoyment of Food, and the single item General Appetite-are associated with higher weight, while the food avoidant sub-scales, Slowness in Eating and Satiety Responsiveness, are associated with lower weight (Kininmonth et al., 2021).

#### 2.1.2 Child Feeding Questionnaire (CFQ)

The widely used Child Feeding Questionnaire (CFQ) (Birch et al., 2001) measures parental beliefs and behaviors regarding their child's feeding and demonstrates good factorial validity (Kong, Vijayasiri, Fitzgibbon, Schiffer, & Campbell, 2015; Liu, Mallan, Mihrshahi, & Daniels, 2014). Perceived Child Weight was assessed with the item ‘How would you describe your son/daughter's weight?’ across a range of child ages (responses: Very underweight, Underweight, Right weight, Overweight, Very overweight). All 25 parents who completed the CFQ reported their perception of their child's weight ‘during the first year of life’ and 14 additionally completed their perception of their child's weight ‘as a toddler’. We assessed Concern about Child Weight using a single item: ‘How concerned are you about your son/daughter's weight at the moment?’ (5-point Likert scale ranging from Unconcerned to Very concerned). We also assessed Pressure to Eat (4 items, e.g., ‘If my child says, ‘I'm not hungry’ I try to get him/her to eat anyway’), Restriction (8 items, e.g., ‘I have to be sure that my child does not eat too many sweet things’) (5-point Likert scale ranging from Disagree to Agree), and Monitoring (e.g. ‘How much do you keep track of the sweet things your child eats?’) (5-point Likert scale ranging from Never to Always). Perceived Responsibility for feeding (e.g., How often are you responsible for deciding what your child's portion sizes are?) (5-point Likert scale ranging from Never to Always) was also assessed.

### 2.2 Comparison to RESONANCE sub-sample

To investigate relationships of parents' perceptions of their child's weight with their actual weight, and parent-report appetite, in infants and toddlers with CF compared with a healthy US population, we used data from a subset of n = 193 infants and toddlers drawn from the RESONANCE cohort. The ongoing longitudinal RESONANCE study examines brain and cognitive development (Chen et al., 2021) as well as measures relevant to growth and appetite (Jansen, Naymik, et al., 2023; Jansen, Thapaliya, et al., 2023), in a community sample of children from infancy through childhood. For the current study we used data on a sub-sample that had anthropometric data collected during infancy and whose parents completed the BEBQ. Parents also completed the single item ‘How would you describe your son/daughter's weight?’ (responses: Underweight, Right weight, Overweight).

### 2.3 Data analysis

Data were analyzed using SPSS 29 software (IBM Corp., Armonk, New York). Z-scores and accompanying percentiles for length, weight, and weight-for-length (WFL) were calculated using World Health Organization, 2006 reference data (World Health Organization, 2006). Furthermore, weight groups of clinical interest were determined following previously described methods (Leonard et al., 2010; Papantoni et al., 2019) such that a WFL of less than the 25th percentile was categorized as poor, 25th-49th acceptable, and 50th and above as the target for young children with CF, with percentiles of 85th or above categorized as ‘increased’. WFL (vs. BMI) was used as it is a current anthropometric standard for children under 2 years old that is used in the Johns Hopkins Pediatric CF Clinic to assess growth, and thus reflects the information on child growth that is given to parents. BEBQ and CFQ subscale scores (including Perceived Child Weight) were calculated as item means with a possible range from 1 to 5. As determined by the Shapiro-Wilks test, all subscales were normally distributed except for satiety responsiveness and the single item assessing general appetite on the BEBQ, and perceived responsibility on the CFQ. Non-parametric tests were therefore used for the latter three constructs. Internal consistency of the BEBQ subscales was determined by Cronbach's alpha with 0.70 or above considered adequate (Tavakol & Dennick, 2011), and Pearson's r or Spearman's rho was used to correlate BEBQ subscale scores with infant and toddler WFL z-scores. Independent samples t-tests and Mann-Whitney U tests were used to compare BEBQ and CFQ subscale means between children perceived underweight (Perceived Child Weight subscale score <3) and children perceived right weight (Perceived Child Weight subscale score ≥3). Based on directional hypotheses supported by previous research in children with CF and healthy children (Kininmonth et al., 2021; Papantoni et al., 2019; Ruzicka, Darling, & Sato, 2021) we implemented one-tailed tests within this small clinical sample, with p < 0.05 deemed statistically significant and p < 0.1 as indicating a statistical trend. Independent samples t-tests and Mann-Whitney U tests were also used to compare BEBQ subscale means between our CF sample and the RESONANCE sub-sample, with two-sided p value < 0.05 deemed statistically significant. Effect sizes to accompany t-tests were calculated as Cohen's *d* with the following interpretation: small (*0.2* ≤ |*d*| *< 0.5),* medium (0.5 ≤ *|d*| < 0.8), and large (|*d|* ≥ 0.8).

## 3 Results

### 3.1 Sample demographics

A total of 32 parents of children <2 years old provided informed consent and entered into the study. Of the 32 total participants, 18 completed both the BEBQ and CFQ, 7 completed only the BEBQ, and 7 completed only the CFQ. Fifteen parents completed the current version of the BEBQ and 10 completed the retrospective version. The sample (N = 32) was predominantly male (72%) and identified as White/Caucasian (84%) in the medical record. Child age ranged between 2 and 24 months with an average age of 11 months. In terms of feeding methods, 11 children were primarily breast/bottle-feeding, 9 were exclusively eating solid foods, and 12 were reported being fed a combination of both. A full description of sample demographics and anthropometric data is given in Table 1 below.

**Table 1:** Sample demographics and anthropometrics for children with CF (n = 32).

Sex (n (%))	Female	9 (28.1%)
	Male	23 (71.9%)
Race (n (%))	White/Caucasian	27 (84.4%)
	Hispanic or Latino	2 (6.3%)
	African American	1 (3.1%)
	More than one race	1 (3.1%)
	Other	1 (3.1%)
Age (months)	10.9 ± 6.3 (2.0–23.2)	
BMI (kg/m^2)^	16.80 ± 1.48 (13.0–19.5)	
Length z-score	−0.73 ± 1.35 (−3.70 – 2.24)	
Weight z-score	−0.34 ± 0.98 (−3.09 – 1.46)	
Weight-for-length z-score	0.16 ± 0.94 (−2.50 – 1.59)	
Weight-for-length percentile	56.26 ± 26.95 (0.62–94.41)	
Genetic mutation (n (%))	F508del/F508del	19 (59.4%)
	F508del/Other	8 (25.0%)
	Other/Other	2 (6.2%)
	Unknown	3 (9.4%)
Pancreatic function status (n (%))	Pancreatic Sufficient	1 (3.1%)
	Pancreatic Insufficient	31 (96.9%)
Gastric tube present (n (%))		1 (3.1%)
Diabetes (n (%))		0 (0%)
Feeding Method (n (%))	Breastfeeding or Bottle-feeding ONLY	11 (34.4%)
	Both breast/bottle-feeding & some solid foods	12 (37.5%)
	Solid foods ONLY	9 (28.1%)

*Footnote:* Values are mean ± standard deviation (range) unless otherwise specified.

### 3.2 Baby Eating Behavior Questionnaire reliability and validity

The BEBQ subscales demonstrated good internal consistency with Cronbach's alpha values ranging from 0.71 to 0.90 (see Table 2). None of the subscales showed significant correlations with WFL z-score.

**Table 2:** BEBQ subscale means, standard deviations, Cronbach's alpha values, and correlations with WFL z-score for children with CF.

BEBQ subscale	Mean (SD)	Cronbach's alpha	Correlation with WFL z-score, *r(p)*
Enjoyment of Food	4.17 (0.67)	0.706	0.01 (0.970)
Food Responsiveness	2.09 (0.63)	0.722	0.09 (0.678)
Slowness in Eating	2.63 (0.97)	0.904	−0.18 (0.933)
Satiety Responsiveness	2.35 (0.83)	0.824	0.18^1^(0.398)
General Appetite	3.80 (1.00)	N/A (1 item)	0.18^1^ (0.379)

*Footnote:* BEBQ = Baby Eating Behavior Questionnaire (scores range from 1 to 5); WFL = weight-for-length; *r* = correlation coefficient; *p* = p-value; ^1^Spearman's rho.

### 3.3 Parental perceptions of weight among children with CF

Parents' perceptions of their child's weight compared to measured weight groups of clinical interest determined based on WFL values relative to WHO (2006) reference data are summarized in Table 3. One child had a WFL under 25th percentile and was correctly perceived as underweight. Of the 11 children between the 25th and 50th percentiles, 5 were perceived as underweight and 6 as the right weight. The remaining 13 children were above the 50th percentile. Of those 13 children, 5 (38%) were perceived as underweight and 8 (62%) as the right weight. No children were perceived as overweight, although 5 children in the over 50th percentile group were above the 85th percentile.

**Table 3:** Parent perceptions of weight by actual weight groups based on measured weight and length among children with CF.

		Perceived Underweight	Perceived Right Weight	Perceived Overweight
**Weight groups**	Under 25th percentile	1 (4%)	0 (0%)	0 (0%)
	25th-50th percentile	5 (20%)	6 (24%)	0 (0%)
	Over 50th percentile	5 (20%)	8 (32%)	0 (0%)

Footnote: Values are number of children (percentage of total sample). Weight groups determined based on WFL values relative to WHO (2006) reference data.

### 3.4 Baby Eating Behavior Questionnaire and Child Feeding Questionnaire scores by Parent perceptions of weight among children with CF

Differences in BEBQ and CFQ scores by parents' perceptions of their child's weight are described in Table 4. Parents who perceived their child as underweight vs. right weight reported greater slowness in eating, with a large effect size, on the BEBQ. The other BEBQ subscale means did not differ significantly by perceived weight; however, the means for enjoyment of food and general appetite were non-significantly lower and satiety responsiveness non-significantly higher for parents who perceived their child as underweight vs. right weight, with large and medium effect sizes.

**Table 4:** BEBQ and CFQ subscale means by perceived weight group among children with CF.

		Perceived Underweight			Perceived Right Weight		
		Mean (SD)	Median (IQR)	Mean (SD)	Median (IQR)	*t* (*p)*	*Cohen's d*
**BEBQ**	Enjoyment of Food	3.93 (0.83)	4.35 (3.25–4.58)	4.30 (0.31)	4.25 (4.00–4.50)	−1.32 (0.103)	−0.625
	Food Responsiveness	1.98 (0.61)	2.20 (1.50–2.58)	1.96 (0.44)	2.00 (1.67–2.17)	0.08 (0.468)	0.038
	Slowness in Eating	3.25 (0.99)	3.00 (2.50–3.88)	2.38 (0.83)	2.25 (1.75–3.00)	**2.04 (**0**.029)**	0.970
	Satiety Responsiveness	2.75 (1.02)	2.33 (1.83–3.00)	2.13 (0.42)	2.00 (1.67–2.67)	33.50^1^ (0.230)	0.828
	General Appetite	3.38 (1.30)	4.00 (2.50–4.00)	4.00 (0.67)	4.00 (3.00–4.00)	057.50^1^ (0.552)	−0.627
**CFQ**	Perceived Child Weight	2.20 (0.51)	2.25 (2.00–2.67)	3.00 (0.00)	3.00 (3.00–3.00)	**−6.13 (<**0**.001)**	−2.388
	Concern about Child Weight	2.45 (1.13)	2.00 (2.00–3.00)	1.50 (0.85)	1.00 (1.00–2.00)	**2.41 (**0**.012)**	0.971
	Perceived Responsibility	4.76 (0.62)	5.00 (5.00–5.00)	4.87 (0.32)	5.00 (5.00–5.00)	79.50^1^ (0.936)	−0.239
	Pressure to Eat	3.73 (0.88)	3,88 (3.13–4.46)	2.97 (1.56)	3.25 (1.13–4.50)	1.45 (0.081)	0.592
	Restriction	2.61 (1.26)	2.38 (1.50–4.00)	2.08 (1.13)	2.13 (1.00–2.71)	0.94 (0.181)	0.446
	Monitoring	4.27 (0.94)	4.67 (3.67–5.00)	3.39 (1.67)	3.67 (1.75–5.00)	1.36 (0.097)	0.703

*Footnote:* BEBQ = Baby Eating Behavior Questionnaire*;* CFQ = Child Feeding Questionnaire (scores on both questionnaires range from 1 to 5); *t* = Between samples t-score; *p* = p-value; ^1^Mann-Whitney U.

Parents who perceived their child as underweight vs. right weight reported greater concern about their weight. The other CFQ subscale means did not differ significantly by perceived weight although there were trends for greater pressure to eat and monitoring, both with medium effect sizes, among parents who perceived their child as underweight vs. right weight.

### 3.5 Comparison to RESONANCE sub-sample

An infant and toddler sub-sample drawn from the RESONANCE cohort (n = 193) was used for comparison. Of this sub-sample, 54.4% were male and the mean age was 7.13 months (S*D* 3.63; range 1.08–16.08 months). The percent male did not statistically differ between samples (χ^2^ = 3.31; *p* = 0.069), but the RESONANCE sample was younger than the sample with CF (*t*(222) = 4.78; *p* = <0.001). The mean WHO WFL z-score for the RESONANCE cohort was 0.53 (*SD* = 1.61) which is higher than the CF sample, but this difference did not reach statistical significance (*p* = .073).

Parental perceptions of child weight were available for n = 176 participants. Parents' perceptions of their child's weight compared to measured weight groups determined based on WFL values relative to WHO (2006) reference data are summarized in Table 5. Of the n = 176 children, 35 children had a WFL under 25th percentile. Of those 35, 83% were perceived as the right weight and 17% perceived as underweight. Of the 22 children between the 25th and 50th percentile, 2 were perceived as underweight and 24 as the right weight. The remaining 115 children had a WFL above the 50th percentile. Of these 115 children 1 (0.9%) was perceived as underweight, 108 (93.9%) were perceived as the right weight, and 6 (5.2%) were perceived as overweight. While only 6 children were perceived as overweight, 59 children had a WFL above the 85th percentile.

**Table 5:** Parent perceptions of child weight by weight groups of clinical interest based on measured weight and length among children in RESONANCE sub-sample.

		Perceived Underweight	Perceived Right Weight	Perceived Overweight
**Weight groups**	Under 25th percentile	6 (3.4%)	29 (16.5%)	0 (0%)
	25th-50th percentile	2 (1.1%)	24 (13.6%)	0 (0%)
	Over 50th percentile	1 (0.6%)	108 (61.4%)	6 (3.4%)

Footnote: Values are number of children (percentage of total sample). Weight groups of interest determined based on WFL values relative to WHO (2006) reference data.

Compared to the RESONANCE sub-sample, children with CF had lower scores on enjoyment of food and higher scores on slowness in eating and a trend toward higher scores on satiety responsiveness on the BEBQ, all with medium effect sizes (see Table 6). Food responsiveness and general appetite did not differ significantly between groups. These results were unchanged when controlling for WFL z-score, age, and sex.

**Table 6:** BEBQ subscale means in infants and toddler with CF compared with RESONANCE sub-sample.

	Children with CF		Children in RESONANCE sub-sample			
	Mean (SD)	Median (IQR)	Mean (SD)	Median (IQR)	*t* (*p*)	*Cohen's d*
Enjoyment of Food	4.17 (0.67)	4.25 (3.88–4.58)	4.55 (0.45)	4.75 (4.25–5.00)	**−3.75 (<**0**.001)**	−0.798
Food Responsiveness	2.09 (0.63)	2.00 (1.67–2.50)	2.02 (0.74)	1.83 (1.50–2.50)	0.43 (0.670)	0.091
Slowness in Eating	2.63 (0.97)	2.50 (1.88–3.25)	2.23 (0.67)	2.25 (1.75–2.63)	**2.61 (**0**.010)**	0.556
Satiety Responsiveness	2.35 (0.83)	2.00 (1.67–2.67)	2.00 (0.64)	2.00 (1.67–2.33)	1831.00^1^ (0.051)	0.518
General Appetite	3.80 (1.00)	4.00 (3.00–4.50)	3.96 (0.94)	4.00 (3.00–5.00)	2557.00^1^ (0.513)	−0.166

*Footnote:* BEBQ = Baby Eating Behavior Questionnaire (scores range from 1 to 5); *t* = Between samples t-score; *p* = p-value; ^1^Mann-Whitney U.

## 4 Discussion

We sought to investigate parental perceptions of weight and appetite, as well as parent feeding behaviors, in a sample of young children with CF, for whom weight and nutritional status are of high clinical relevance.

The BEBQ demonstrated good internal consistency on all four subscales in this sample of 32 young children with CF. These findings suggest that the BEBQ could be a useful clinical tool for parents to report on appetite during the milk-feeding stage among children with CF who are under two years of age. In ostensible contrast to our earlier findings in children with CF who were 2–12-year-olds for which we used the CEBQ (Papantoni et al., 2019), and to established relationships in healthy pediatric populations (Kininmonth et al., 2021), BEBQ subscale scores did not significantly correlate with child WFL z-scores. One explanation for the lack of significant correlation of other BEBQ sub-scales and WFL z-score is that these appetitive traits may not yet have translated into differences in weight during this early stage of life. Alternatively, individual differences in weight may not yet have influenced parents' perceptions of weight and thereby contributed to their perceptions of child appetite. We note that the lack of correlation between BEBQ subscales and weight is somewhat consistent with our hypothesis and interpretation that parents of children with CF are particularly concerned with their child's weight and appetite status. Therefore their perceptions of their child's appetite may be disproportionately reflective of their concerns about their appetite as compared to children's actual, weight-associated appetitive tendencies. Nevertheless, slowness in eating was negatively correlated with WFL z-score (r = −0.18), and general appetite was positively correlated with WFL z-score (rho = 0.18). These appetite measures therefore demonstrated the expected directions of relationships with body weight, although we note that the relationship for slowness in eating was only half of the strength of the relationship between that scale and BMI that we observed in older children with CF (Papantoni et al., 2019). The strong internal consistency of the subscale items provides some assurance that parents of children with CF were able to understand and coherently complete the items, and increases our confidence that the pattern of results we observed was not driven by inadequacy of the measures used.

Parents of young children with CF demonstrated high rates of perceived underweight, despite the relatively low occurrence of weights that would be considered low enough to merit clinical concern. Nearly half of the sample was perceived as underweight, and no children were perceived as overweight. Yet only one child had a WFL under the 25th percentile, and one-fifth of children were over the 85th percentile. This pattern was strikingly different from the community sample examined, in which only 4.5% of children were perceived as underweight while 91% of were perceived to be the right weight, and in which 10% of children over 85th percentile were perceived as overweight. These data, which are consistent with previous qualitative literature demonstrating that parents of children with CF are particularly worried about their children being underweight (Savage & Callery, 2005), suggest that parents of infants and toddlers with CF are primed to perceive their child as underweight even if they are healthy weight. They further suggest that parents of children with CF may be even more likely than parents of children without CF to underestimate the weight of children whose WFL places them at potential risk for overweight and associated sequelae. We also found that, compared to a US community sample, parents of infants and toddlers with CF reported lesser child enjoyment of food and greater slowness in eating, as well as a trend toward greater satiety responsiveness, with medium effect sizes, even when controlling for any sample differences in WFL z-scores, age and sex. These findings support further investigation of biobehavioral mechanisms underlying lower appetite in young children with CF which could potentially benefit populations with CF, and also populations without CF, by improving understanding of mechanisms that could be harnessed to either boost, or reduce, appetite as clinically indicated.

Parents who perceived their child as underweight expressed greater concern regarding their child's weight and reported greater slowness in eating, with large effect size, compared to parents who perceived their child as the right weight. The presence of these associations of appetitive traits with parents' weight perceptions in the absence of significant associations with actual child weight status is consistent with a model in which some parents of infants and toddlers with CF perceive their children as low in both weight and appetite even though weights are not in fact low enough to merit clinical concern. Since the BEBQ is a parent-report measure we cannot confidently conclude that children whose parents reported lower appetite indeed had lower appetite if measured objectively. However, this interpretation is supported to some degree by evidence that BEBQ scales show cross-sectional and prospective associations with body weight in mostly clinical samples (Kininmonth et al., 2021), and that appetitive traits as assessed by the CEBQ are associated with weight (Kininmonth et al., 2021), and with objective measures of eating behavior (Carnell & Wardle, 2007). Our findings could, therefore, reflect that there is a group of young children with CF who show reduced appetite which is observed by parents and colors the parent's perception of the child's weight in concert with their observations of the child's actual growth. Alternatively, there may be a group of parents who show heightened sensitivity to poor growth and reduced appetite in their child which is reflected in parent-report measures and may not show a tight correspondence with actual appetite or weight problems.

Our exploratory analyses of differences in child feeding behaviors by parent weight perceptions did not reveal statistically significant phenomena. However, the trend for increased pressure to eat with perceived underweight vs. right weight was consistent with a wealth of literature in healthy populations showing higher pressure to eat with lower perceived child weight (Wang, Winkley, Wei, Cao, & Chang, 2023). In contrast, the trend in the current sample for increased monitoring with perceived underweight vs. right weight is somewhat in opposition with literature in healthy populations which shows higher rates of monitoring in parents who express concern about future *over*weight (Seburg et al., 2014). This represents an important potential point of differentiation between the reasons that motivate monitoring of intake of high-fat and other foods and possibly the goals of these monitoring behaviors among clinical samples of parents of children for whom under-rather than over-nutrition is the prominent concern. However, since diet and specific monitoring goals were not measured in the current study, we are unable to tell whether higher monitoring scores reflect parents’ efforts to limit or promote intake of sweet/high-fat/snack-foods in their young children; this would be an important topic for future investigation.

This cross-sectional study had several limitations. The sample size was small and mostly consisted of male children whose parents identified as white, which may limit the generalizability of the sample to the entire population of infants and toddlers with CF. While the proportion of males was higher than usual in the age cohort targeted for recruitment from the CF clinic, the majority of the group agreed to participate in the study which provides some reassurance that our results were not affected by sampling bias. Further, our assessments of parent weight perceptions may have underestimated parents' understanding of their child's risk of overweight. For example, we found in a previous study that although few parents perceived their overweight children as overweight, a majority expressed concern about their overweight child becoming overweight in the future (Carnell, Edwards, Croker, Boniface, & Wardle, 2005). The BEBQ and CFQ are parent report measures vulnerable to bias that may not reflect true appetitive characteristics of the infants and toddlers and we therefore interpret our data as representing parent perceptions of child appetite and feeding interactions rather than objective measures of eating and feeding behavior. Additionally, the CFQ is primarily used for children over the age of 2. The CFQ questions related to feeding practices-such as restriction, pressure, and monitoring-may not be as age-appropriate for the milk-feeding only children as compared to those eating solid foods. These limitations notwithstanding, our results demonstrate the potential utility of the BEBQ in a clinical sample. Further, they suggest it may be helpful for clinicians to educate parents of young children with CF on normative growth trajectories and to explore parents' perceptions of child weight and appetite and parent-child feeding interactions so as to promote a fuller understanding of the child's nutritional status, facilitate appropriate feeding behaviors and alleviate unnecessary concerns.

## Funding

This project was funded by NIH grant R01DK113286 with additional support for the team from and UH3OD023313 and BMGF grant INV-006627.

## Ethical statement

This study was approved by the Johns Hopkins University School of Medicine Institutional Review Board.

## CRediT authorship contribution statement

**Sarah Ann Duck:** Writing – review & editing, Writing – original draft, Visualization, Formal analysis, Data curation, Conceptualization. **Elena Jansen:** Writing – review & editing, Data curation. **Afroditi Papantoni:** Writing – review & editing, Investigation. **Aerial Sheltry:** Writing – review & editing, Investigation. **Daphne Koinis-Mitchell:** Resources. **Viren D’Sa:** Resources. **Sean Deoni:** Resources. **Timothy H. Moran:** Writing – review & editing. **Robert L. Findling:** Writing – review & editing. **Peter J. Mogayzel:** Writing – review & editing, Supervision, Resources, Conceptualization. **Susan Carnell:** Writing – review & editing, Supervision, Funding acquisition, Conceptualization.

## Declaration of competing interest

Dr. Findling receives or has received research support, acted as a consultant and/or has received honoraria from Abbvie, Acadia, Adamas, Afecta, Ajna, Akili, Alkermes, American Academy of Child & Adolescent Psychiatry, American Psychiatric Press, Bioprojet, BioXcel, Corium, Elsevier, Idorsia, Iqvia, Karuna, Lundbeck, Merck, MJH Life Sciences, NIH, Neurim, Novartis, Otsuka, Oxford University Press, PaxMedica, PCORI, Pfizer, Physicians’ Postgraduate Press, Radius, Receptor Life Sciences, Sage, Signant Health, Sumitomo Pharma, Sunovion, Supernus Pharmaceuticals, Syneos, Takeda, Tris, and Viatris. Dr. Mogayzel has received research support Vertex Pharmaceuticals, Eloxx Pharmaceuticals, Astra Zeneca, and 4DMedical. Dr. Carnell has received funding from Eli Lilly and Company. Sean C. L. Deoni has relationships with Nestlé Research, Nestlé Nutrition, Wyeth Nutrition, and Mead Johnson Nutrition.

The other authors have no conflicts of interest to disclose.

This project was funded by NIH grant R01DK113286 with additional support for the team from and UH3OD023313 and BMGF grant INV-006627.

## Data Availability

Data will be made available on request.
