# Supplementary material for: Parental perceptions of body weight and appetite in infants and toddlers with cystic fibrosis
Source: Appetite. 2024 Jul 1;198:107357. doi: 10.1016/j.appet.2024.107357 (PMC11134312; doi:10.1016/j.appet.2024.107357)
Supplement: Multimedia component 1 [file mmc1.docx]

*Supplementary Figure 1. BEBQ subscales in children with CF compared with children from RESONANCE sub-sample*


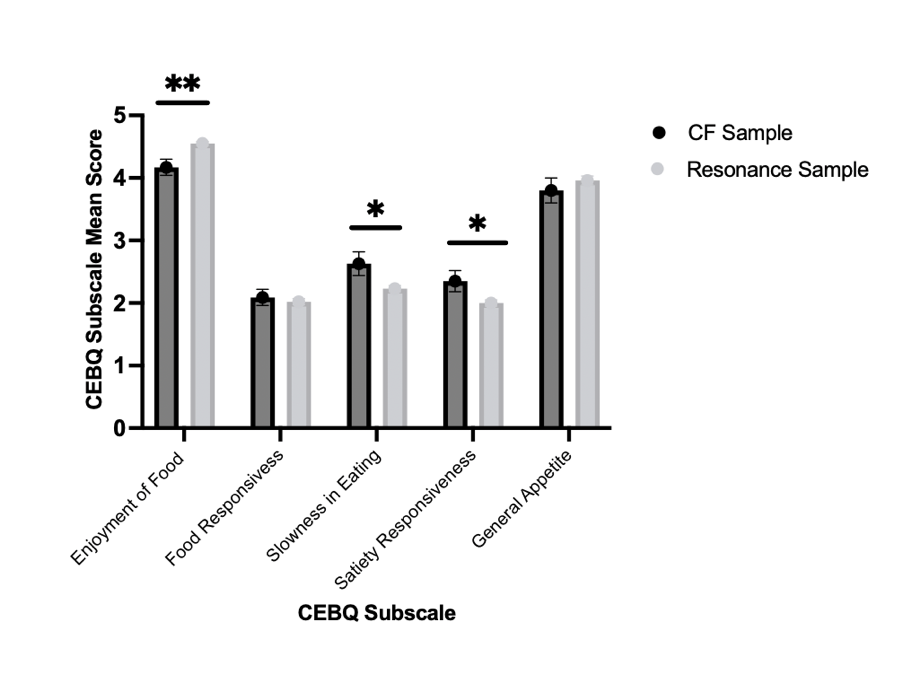


*Footnote: * p<0.05, ** p<0.001. Bars represent means with standard error.*
